# Durum Wheat (*Triticum Durum* Desf.) Lines Show Different Abilities to Form Masked Mycotoxins under Greenhouse Conditions

**DOI:** 10.3390/toxins6010081

**Published:** 2013-12-24

**Authors:** Martina Cirlini, Silvia Generotti, Andrea Dall’Erta, Pietro Lancioni, Gianluca Ferrazzano, Andrea Massi, Gianni Galaverna, Chiara Dall’Asta

**Affiliations:** 1 Department of Food Science, University of Parma, Parco Area delle Scienze 95/A, Parma 43124, Italy; E-Mails: martina.cirlini@unipr.it (M.C.); andrea.dallerta@unipr.it (A.D.); gianni.galaverna@unipr.it (G.G.); 2 Barilla G. R. F.lli SpA, Food Research Labs, Parma 43124, Italy; E-Mail: silvia.generotti@barilla.com; 3 Società Produttori Sementi Spa, Via Macero 1, Argelato 40050, Italy; E-Mails: pietro.lancioni@sygenta.com (P.L.); g.ferrazzano@prosementi.com (G.F.); a.massi@prosementi.com (A.M.)

**Keywords:** masked mycotoxins, fusarium head blight, pasta, deoxynivalenol, virulence factor

## Abstract

Deoxynivalenol (DON) is the most prevalent trichothecene in Europe and its occurrence is associated with infections of *Fusarium graminearum* and *F. culmorum*, causal agents of Fusarium head blight (FHB) on wheat. Resistance to FHB is a complex character and high variability occurs in the relationship between DON content and FHB incidence. DON conjugation to glucose (DON-3-glucoside, D3G) is the primary plant mechanism for resistance towards DON accumulation. Although this mechanism has been already described in bread wheat and barley, no data are reported so far about durum wheat, a key cereal in the pasta production chain. To address this issue, the ability of durum wheat to detoxify and convert deoxynivalenol into D3G was studied under greenhouse controlled conditions. Four durum wheat varieties (Svevo, Claudio, Kofa and Neodur) were assessed for DON-D3G conversion; Sumai 3, a bread wheat variety carrying a major QTL for FHB resistance (QFhs.ndsu-3B), was used as a positive control. Data reported hereby clearly demonstrate the ability of durum wheat to convert deoxynivalenol into its conjugated form, D3G.

## 1. Introduction

Fusarium head blight (FHB) is one of the most deleterious fungal diseases affecting wheat worldwide: it is related to infection by pathogenic fungi of the *Fusarium* spp. and it is widely diffused, especially in those areas with inductive climatic conditions (hot/warm temperatures and high/medium high humidity) [1,2,3]. FHB causes severe yield losses (up to 70%), affecting the quality of grains which show low protein content and color defects. Moreover, fungal infection may lead to the accumulation of mycotoxins: depending on the chemotype of the fungus, the type B trichothecenes deoxynivalenol (DON), nivalenol, 3-acetyldeoxynivalenol (3-ADON), and 15-acetyldeoxynivalenol (15-ADON) often accumulate in the developing grain [4].

This contamination is especially critical for durum wheat, which is used primarily for human consumption. Although the best economic and ecological strategy for reducing FHB damage is the utilization of resistant cultivars, the attempts to define durum wheat resistant lines have been unsuccessful so far.

In soft wheat, numerous QTL related to FHB resistance have been described [5]. In particular, several studies performed using the high resistant Chinese Spring wheat line Sumai-3 showed that the two most effective QTLs related to FHB resistance are positioned on the short arm of chromosome 3B (*Fhb1*) and on chromosome 5A (*Qfhs.ifa-5A*) [6,7,8].

Durum wheat cultivars were generally considered to be susceptible to FHB. Indeed, no variation in resistance to FHB has been found within *T. durum* lines, even among large germplasm collections of several thousand lines [9,10].

This fact may be due to several factors: a narrow genetic base compared to hexaploid wheat might be linked to the fact that durum wheat is tretraploid and that limited breeding efforts have been undertaken on this crop [11]. Attempts to transfer resistance from hexaploid into tetraploid wheat have been met with limited success [12,13].

Several types of resistance to FHB are known in wheat, classified as type I (resistance towards initial infection of spikelets), type II (resistance against spread of pathogen within spike) [14,15] and type III (resistance to DON accumulation in grains) [16,17].

In particular, DON was proven to inhibit protein synthesis in eukaryotic cells and acts as a virulence factor during fungal pathogenesis, therefore resistance to DON is considered an important component of resistance against FHB [18]. As reported by several studies, one mechanism of resistance to DON is the conversion of DON into the less toxic metabolites deoxynivalenol-3-*O*-glucoside (D3G) [19,20,21]. In particular, in a wheat population segregating for Fhb1, lines containing the Fhb1 resistance allele efficiently conjugate DON to the less toxic D3G [19]. In this work, the authors reported a good correlation between FHB resistance and DON conversion rate, expressed as [D3G]/[DON] ratio. This hypothesis was recently questioned by Gunnaiah *et al*. [22], whose study showed that DON resistance is not a major mechanism of FHB resistance associated with Nyubai Alleles of *Fhb1*.

The co-occurrence of DON and D3G in durum wheat harvested in Northern Italy was recently reported [23], showing a diffuse contamination of most samples with both compounds present at significant levels.

The present work is aimed at the study of the DON-to-D3G conversion ability of wheat lines (four durum wheat genotypes and one soft wheat genotype) under controlled greenhouse conditions.

In particular, the soft wheat line Sumai-3 was chosen as reference standard based on its well-known resistance towards FHB [24,25,26], while durum wheat lines (Kofa, Svevo, Neodur and Claudio) were chosen on account of their technological performances and because have been widely used in the most relevant durum breeding programs.

In a first trial, plants have been treated with *F. graminearum* and with DON under controlled growing conditions and samples have been analyzed for free and masked mycotoxins content. Then, those lines that had showed strong differences in the first trial were further considered in a second trial, just focusing on the DON-treatment at anthesis.

## 2. Results and Discussion

### 2.1. Set up of the First Trial

**Figure 1:**
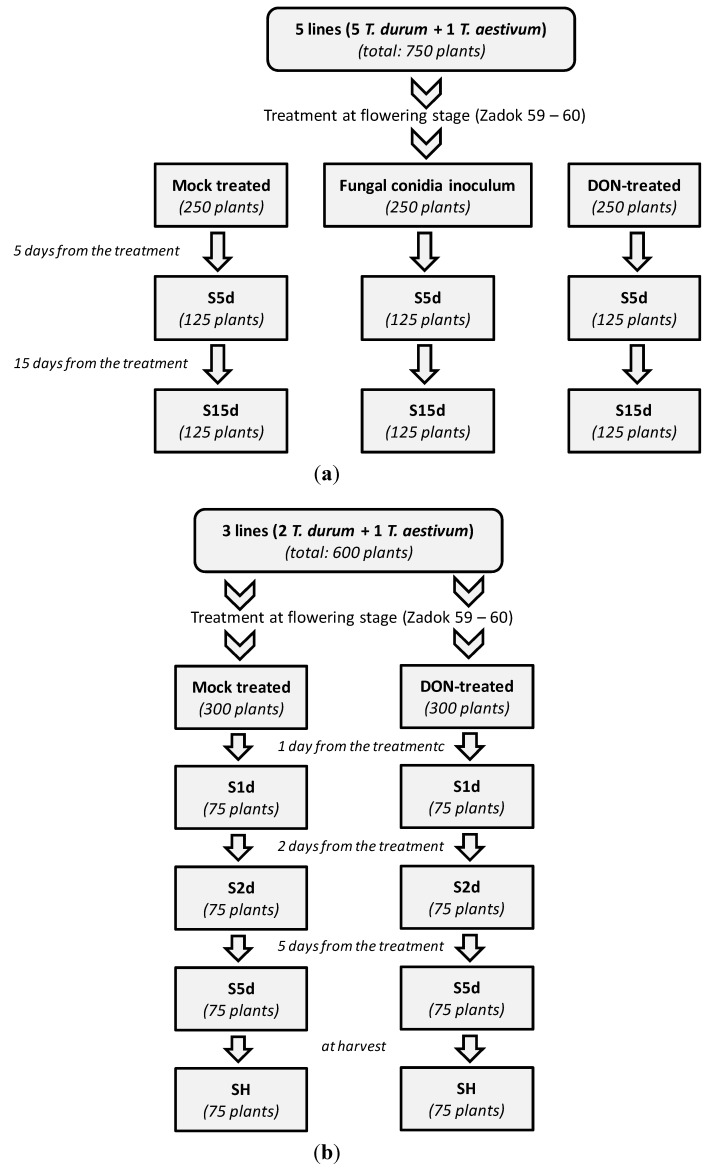
Experiment set up: (**a**) first trial; (**b**) second trial.

Two different trials were performed within this study, following the general scheme reported in Figure 1. The first trial involved four genotypes of *Triticum durum* (Kofa, Svevo, Claudio, Neodur) and one genotype of *T. aestivum* (Sumai-3), selected on the basis of their resistance towards Fusarium head blight (FHB) under in-field conditions and their genetic background. In particular, the DON detoxification ability related to FHB resistance was largely studied in soft wheat [27], while very few studies only reported the occurrence of D3G in durum wheat under natural infection conditions, so far [23].

We decided to compare contamination data obtained upon fungal conidia inoculation and DON-treatment, in consideration of the possible different effects pointed out by these treatment. In particular, the inoculation with *F. graminearum* at the flowering stage (Zadoks 60) [28] easily resembles the plant infection occurring in field, causing thus a similar plant-pathogen cross-talk. On the other hand, the direct treatment with DON, although extremely simplified compared to the natural phenomenon, allows to maximize those enzymatic mechanisms carried out by the plant to limit the DON toxic action during fungal infection.

Plants (*n* = 750) were split into three groups and underwent different treatments at the flowering stage, namely inoculation with fungal conidia suspension, treatment with DON solution and mock-treatment with distilled water (control group). Each group was sampled after five days and after 15 days from the treatment (see Figure 1a). Sampling times were chosen in order to highlight the DON detoxification ability in the first phases after the fungal infection, since reports published until now commonly focused on samples collected at harvest (full maturation stage) [19].

#### 2.1.1. Fusarium graminearum *Treated Samples*

In the first trial, four selected durum wheat lines (Kofa, Svevo, Claudio and Neodur) and a soft wheat line (Sumai-3) were inoculated with *F. graminearum*, following the scheme reported in Figure 1a.

Since also other trichothecenes could be present besides DON and D3G, acetylated-DON derivatives as well as other type B and type A trichothecenes were also screened.

No detectable contamination was found in the control spikes, thus ensuring the reliability of the experiment, while all treated samples were found to be positive for both DON and D3G.

Acetylated-DON were found in most of the samples collected at S15d, while other type B and type A trichothecenes were not detected at any stage. Since the fungal growth was not comparable in the considered samples, the ergosterol content was used for data normalization. Ergosterol can be efficiently used as fungal growth biomarker as the artificially inoculated fungal strain was the only one responsible for the fungal biomass in the considered system.

Total contamination expressed as the sum of DON and analogues at S5d was in the range 4.8–17.4 mg/Kg, thus indicating a significant mycotoxin production already after five days from the fungal inoculation. Besides Claudio that showed a significantly higher toxin amount, the other wheat lines were found to be comparable in terms of total contamination. D3G was found in all the considered samples at levels ranging from 0.2 to 4.4 mg/Kg, with an average [D3G]/[DON] ratio of about 0.3.

Since significant differences between lines were not outlined at this stage, results obtained at S15d were better considered for the discussion.

Total contamination at S15d, expressed as the sum of DON, D3G and ADON, ranged from 49 to 168 mg/Kg, as reported in Table 1, thus indicating that the trend obtained at S5d stage was confirmed also after 15 days from the fungal inoculation.

**Table 1:** Total contamination (TD) expressed as the sum of deoxynivalenol (DON), deoxynivalenol-3-*O*-glucoside (D3G), acetyldeoxynivalenol (ADON) (mg/Kg) and ergosterol content determined in *F. graminearum* treated wheat lines at S15d stage. Data are referred to the first trial, described in Figure 1a.

Cultivars	TD (mg/Kg)	Ergosterol (mg/Kg)
Claudio	168 ± 34	22.0 ± 7.7
Neodur	90 ± 20	5.2 ± 1.8
Kofa	79 ± 5	4.0 ± 1.3
Svevo	129 ± 6	10.1 ± 0.7
Sumai-3	49 ± 6	2.5 ± 0.2

Among durum wheat varieties, Claudio and Svevo showed the highest contamination at S15d, while concentrations found in Neodur and Kofa were comparable. According to the ergosterol content, Claudio supported also the highest fungal growth compared to other lines.

**Figure 2:**
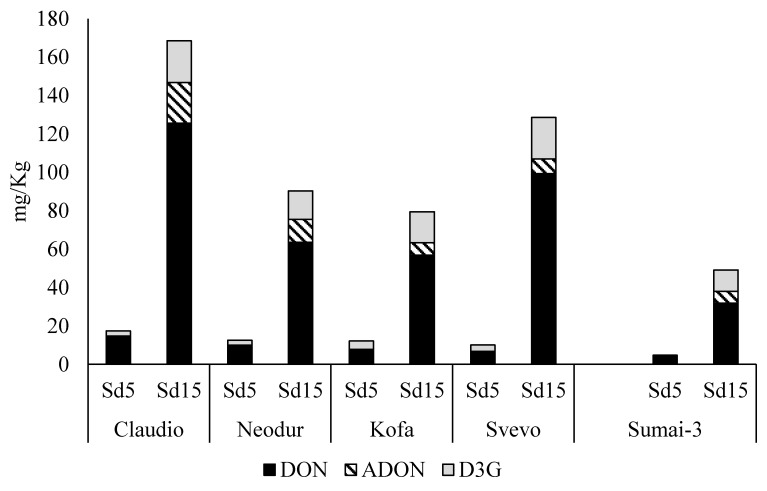
DON, ADON and D3G amount found in *F. graminearum* treated samples collected at S5d and S15d stages. Data referred to the first trial, described in Figure 1a.

Data, normalised on the ergosterol content and expressed as relative percentage of DON and D3G found in the five biological replicates considered for both maturation stages, are reported in Figure 2.

At first, from the plot, Sumai-3 showed the lower toxin accumulation compared to the durum wheat lines. This result is supported by a lower fungal growth compared to the other lines, measured as ergosterol content (see Table 1). These data are in agreement with the literature, since Sumai-3 is often reported as a highly resistant line towards FHB and DON accumulation [29,30]. Furthermore, the strongest toxin accumulation after 15 days from the inoculum was recorded for Claudio cv. also in this case in agreement with ergosterol data.

Concentration ratio values obtained for durum wheat lines and expressed as the ratio between D3G and DON concentrations, were statistically compared by ANOVA analysis followed by a *post-hoc* Tuckey test; results are reported in Table 2. The sampling time was found significant for all the considered samples (*p* = 0.000), thus indicating that the DON conversion starts after the treatment and progressively increases during the maturation. Kofa and Svevo showed a good conversion rate (0.25 and 0.28, respectively), while [D3G]/[TDON] ratio was the lowest in Claudio (0.14).

**Table 2:** Conversion rate expressed as [D3G]/[TDON] (where TDON is the sum of DON, D3G and ADON) found in durum wheat lines at S15d stage. Different letters indicate significantly different values. Data are referred to the first trial, described in Figure 1a.

Cultivars	[D3G]/[TDON]	
	*DON-Treated Samples*	*F. graminearum Inoculated Samples*
Claudio	0.292 b	0.144 b
Neodur	0.371 a	0.182 b
Kofa	0.407 a	0.252 a
Svevo	0.273 b	0.282 a

#### 2.1.2. DON-Treated Samples

Besides fungal inoculation, wheat lines were also treated with DON, as already performed by Lemmens *et al*. [19]. The use of a simplified system, although very far from the complicated cross-talk phenomena occurring upon in field fungal inoculation, allows distinguishing the possible differences in the transformation occurring in plants. Differently from Lemmens *et al*. [19], we decided to apply an amount of DON 10-times lower (total amount: 80 µg) to better resemble the common conditions experimented under in-field infection.

All the DON-treated and control spikes were separately analysed for DON and D3G content; each analysis was performed in duplicate.

**Figure 3:**
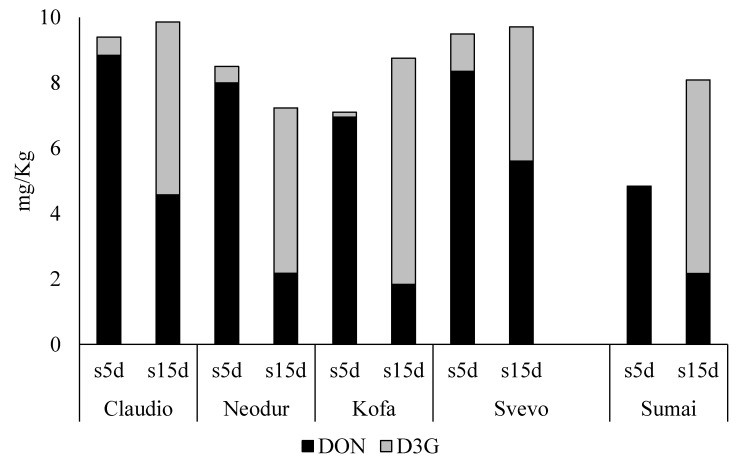
DON and D3G concentrations found in DON-treated samples collected at S5d and S15d stages. Data are referred to the first trial, described in Figure 1a.

As already reported for *F. graminearum* treated plants, no detectable DON contamination was found in the control spikes, while all DON-treated samples were found to be positive for both DON and D3G. Data, expressed as concentration of DON and D3G found in the five biological replicates at both maturation stages, are reported in Figure 3.

As a general comment, all the conversion rates observed in the DON-treated samples were extremely higher than those reported for plants treated with *F. graminearum*. This could be explained considering two points: (a) at first, the toxin concentration levels reached in *F. graminearum* treated samples are definitely higher than those obtained in the DON-treatment; (b) then, the fungal growth under inductive conditions was strong, with severe pathogenesis symptoms. In consideration of these conditions, it is reasonable to deduce that the detoxification occurred at a lower extent.

D3G was found to be higher or comparable to DON at S15d stage in all the considered lines, but an important conversion was already noticed five days from the treatment (S5d).

The *Sumai-3* line confirmed the strong DON-to-D3G conversion ability already 15 days from the toxin treatment, being the ratio between D3G and total DON-related compounds (TDON) 0.731 at S15d.

Among durum wheat lines, Kofa showed the highest conversion ability, as reported in Table 2; on the other hand, Svevo showed the lowest conversion rate. It should be noticed that DON concentration was found higher than D3G only in Svevo samples.

As further observation, the total DON concentration at S15d was higher in Claudio and Svevo than in Neodur and Kofa, thus suggesting that possible other conjugation mechanisms may be active in plant.

In addition, differently from other lines, Svevo and Claudio also presented comparable total DON concentration at S5d and S15d: also in this case, lower bioconversion ability could be considered as a possible explanation.

Interestingly, our results clearly showed that D3G formation *in planta* already started five days from the treatment.

Similarly to the results obtained for *F. graminearum* treated samples, Kofa was found to show the highest conversion rate; on the contrary, Svevo showed a significantly higher conversion rate upon fungal inoculation compared to what was observed upon DON-treatment. This fact could be due to the absence of fungal growth, and should be carefully considered in further studies.

### 2.2. Set up of the Second Trial

Starting from the results obtained in the first trial, a second experiment was set up. According to the data obtained, Kofa and Svevo durum wheat lines showed the most different detoxification ability and thus were selected for further investigation, in comparison to Sumai-3.

Plants (*n* = 600) were split into two groups and alternatively underwent DON-treatment and mock-inoculation (control group) at the flowering stage (see Figure 1b).

DON-treated plants were compared to control group by collecting samples one day (Sd1), two days (Sd2), and five days (Sd5) from the treatment, and at harvest. A recent study reported that UDP-glucosyltransferase proteins in Sumai-3, involved in the DON detoxification process, are over-expressed already 32–48 h from the fungal inoculation [29]; thus, short times after treatment were considered to better understand the D3G formation at the early-stage. In addition, spikes at full maturation were harvested and analysed to evaluate the final content of DON and D3G.

#### DON-Treated Samples

The general trend observed in the first trial was confirmed, although this time the conversion rate at harvest was lower for the considered lines, as reported in Table 3. This fact may be due to the inter-individual differences between plants and to the slightly different environmental conditions not directly dependent from the controlled agronomical parameters (e.g., external climatic factors), experimented in the greenhouse during the trials.

**Table 3:** Conversion rate expressed as [D3G]/[TDON] (where TDON is the sum of DON, D3G and ADON) obtained at each sampling stage for the DON-treated lines considered in the second trial.

Sampling dates	Kofa	Svevo	Sumai-3
Sd1	0.046	0.056	0.246
Sd2	0.391	0.154	0.655
Sd5	0.421	0.115	0.619
SH	0.530	0.312	0.843

Conversion rate values were statistically compared by ANOVA followed by *post-hoc* Tuckey test. The sampling stage was found to be significant for all the considered lines (*p* = 0.000). D3G was found in all the analysed samples already after 24 hours from the treatment, thus indicating that the detoxification pathway is promptly active towards DON in plant. This fact is in agreement with the expression data recently reported by Gottwald *et al*. [29] for FHB resistant soft wheat lines, among those Sumai-3. In particular, the authors stated that the expression profiles of genes related to detoxification processes in resistant genotypes are inducted in the early stage, while susceptible lines typically show late inductions.

As reported in Table 3, Sumai-3 showed the highest conversion rate already after 24 h from the treatment and its detoxification ability is confirmed as very high at harvest. Concerning durum wheat, also in this second trial, Kofa showed a higher detoxification ability compared to Svevo already after two days from the treatment (*p* = 0.003 at Sd2) and this difference became particularly evident at harvest. The data here presented are clearly in agreement with Gottwald *et al*. [29], since Svevo, which is usually assumed to be less resistant than Kofa towards FHB, showed a delayed conversion of DON to D3G.

## 3. Experimental Section

### 3.1. Chemicals

Sodium acetate, formic acid, methanol, hexane, ethyl acetate, bis-silyltrifluoroacetamide (BSTFA) and acetonitrile were purchased from Sigma-Aldrich (Taufkirchen, Germany). All solvents were HPLC grade. Ultra-pure water was in-house produced by using a Milli-Q System (Millipore, Bedford, MA, USA). The following mycotoxin standard solutions were purchased from RomerLabs (Tulln, Austria): ^13^C-Deoxynivalenol (internal standard, 25 mg/L in acetonitrile), D3G (50 mg/L in acetonitrile), mix A + B trichothecenes containing DON, NIV, T-2 and HT-2 (10 mg/L each in acetonitrile). All standard solutions were stored at −20 °C and brought to room temperature before use. A working solution (2.5 µg/mL) containing all the target mycotoxins was prepared in water/methanol (80/20, v/v) by combining and properly diluting suitable aliquots of each standard. Ergosterol and cholestanol were purchased from Sigma-Aldrich (Taufkirchen, Germany). *F. graminearum* strain was purchased from Department of Agronomy (DiPSA) University of Bologna.

### 3.2. Wheat Lines Background Information

For this study, four durum wheat lines (Kofa, Svevo, Neodur and Claudio) and one soft wheat genotype (Sumai-3) have been chosen.

Kofa, is a Southwestern United States cv. released by Western Plant Breeders (Arizona) obtained from a population based on multiple parents (dicoccum alpha pop-85 S-1) mainly related to the American and CIMMYT germplasm, with the inclusion of emmer accessions. Svevo, an Italian cv. released by Società Produttori Sementi, has been obtained from the cross between a CIMMYT line (pedigree rok/fg//stil/3/dur1/4/sapi/teal//hui) related to the cv. Yavaros, genetic background (Jori/Anhinga//Flamingo), and the cv. Zenit originating from a cross between Italian and American accessions (Valriccardo/Vic). Both Kofa and Svevo are well adapted to the Mediterranean climate and can be classified as early-flowering genotypes in such conditions, and are susceptible to FHB. Neodur (pedigree 184-7/Valdur//Edmore) is a late flowering variety cultivated in north Italy carrying a major QTL for resistance against the soil-borne cereal mosaic virus. Claudio (pedigree Sel.Cimmyt35/Durango//ISEA1938xGrazia) is a medium late variety widely cultivated in Italy and France well known for its yield stability. Both Neodur and Claudio can be considered moderately tolerant to FHB under Mediterranean growing conditions.

The four elite varieties of durum wheat (Svevo, Claudio, Kofa e Neodur) were selected for a preliminary study on the ability to convert DON into D3G. Lines were initially chosen based on the different susceptibility to DON accumulation and to FHB symptoms, Kofa and Svevo showing medium susceptibility and Claudio and Neodur medium tolerance. Moreover, the selected lines well represent the genetic diversity of the main ameliorated pool of durum wheat adapted to grow in the Mediterranean area. The soft wheat cv. Sumai 3 was used as control for its known resistance ability towards FHB (type II resistance, linked to the QTL *QFhs.ndsu-3B*).

### 3.3. Fusarium graminearum Strain Background Information

*Fusarium graminearum* strain F566 was used for plant inoculation; the strain was isolated from wheat samples harvested in Northern Italy (Bologna area, Emilia Romagna region); the chemotype was fully characterized as reported by Prodi *et al*. [30], as well as its aggressiveness [31]. The strain is conserved in the Phytopatological Mycology Collection of the Department of Agricultural Sciences of the University of Bologna.

### 3.4. Design of the Greenhouse Experiment

The artificial inoculation experiment on durum wheat plants has been carried out according to a RCB (Randomized Complete Block) scheme within a greenhouse plant at Produttori Sementi S.p.A. (Argelato, Bologna, Italy). All the plants were singularly sown and maintained at low temperature (5 °C) for 30 days to mimic vernal conditions (vernalization), then they were transferred into the greenhouse, fixing the ambient conditions in order to mimic first the spring period and then the summer season. For the first experiment, four lines of durum wheat (Svevo, Claudio, Kofa e Neodur) and one line of soft wheat chosen as a reference line for its resistance to FHB (Sumai 3) were selected.

For each line, four groups were considered, each formed by five plants: at the flowering stage (zadok 59–60) the first group was artificially inoculated with a *F. graminearum* inoculum, the second group was contaminated with a DON solution, whereas the third group was considered as the control one. All the contaminated groups, as well as the negative (not contaminated) control, were replicated five times. The entire experiment was performed in triplicate in order to allow two different sampling steps at two different maturation stages. Sampling steps were fixed from the inoculation step as follows: after five days (S5d) and after 15 days (S15d). The total plant number was 750. The treatment was performed on four spikelets in the central part of the spike: each spikelet was inoculated with 10 µL of a fungal conidia solution (1 × 10^5^ conidia/mL) or with 20 µL DON standard solution (exact title: 0.828 mg/mL). The control samples were mock treated with distilled water.

The second trial was focused on two lines of durum wheat (Svevo, and Kofa) and one line of soft wheat (Sumai 3). In this case, four sampling steps at four maturation stages were considered. Sampling steps were fixed from the treatment step as follows: after one day (S1d), after two days (S2d), after five days (S5d) and at harvest (SH). The total number of plants was 600. The treatment with DON solution was performed as above. Control plants were mock-treated with distilled water.

At each sampling stage, spikes were separately collected and milled, before LC-MS analysis. For fungal contaminated samples, data were normalized on the amount of fungal biomass developed, using ergosterol as fungal growth biomarker.

### 3.5. Free and Masked Mycotoxins Analysis

Samples were prepared according to Berthiller *et al*. [32] with slight modifications. Briefly, the whole ears were finely grounded and mixed 0.5 g of grounded wheat was extracted for 90 min at 200 strokes/min on a shaker with 2 mL of acetonitrile/water (80/20, v/v) acidified with 0.1% of formic acid. The extract was collected and centrifuged for 10 min at 1,4000 rpm at room temperature, then 1 mL was evaporated to dryness under nitrogen. After addition of the internal standard (^13^C-DON, 20 µL), the residue was dissolved in 1 mL of water/methanol (80/20, v/v) and analyzed by UPLC-ESI/MS.

The UPLC-ESI/MS analyses were carried out according to Dall’Asta *et al*. [23], using an Acquity UPLC separation system (Waters Co., Milford, MA, USA) equipped with an Acquity Single Quadrupole MS detector with an electrospray source. Chromatographic conditions were as follows: column, Acquity UPLC BEH C18 (1.7 µm, 2.1 × 50 mm); flow rate, 0.35 mL/min; column temperature, 40 °C; injection volume, 5 µL; gradient elution using 0.1 mM sodium acetate solution in water (eluent A) and methanol (eluent B), both acidified with 0.2% formic acid. Gradient conditions: initial conditions were set at 2% B for 1 min, then eluent B was increased to 20% in 1 min; after an isocratic step (6 min), eluent B was increased to 90% in 9 min; after a 3 min isocratic step, the system was re-equilibrated to initial conditions for 3 min. The total analysis time was 23 min. The ESI source was operated in positive ionization mode. MS parameters were as follows: capillary voltage, 2.50 kV; cone, 30 V; source block temperature, 120 °C; desolvation temperature, 350 °C; cone gas, 50 L/h; desolvation gas, 850 L/h. Detection was performed using single ion monitoring mode and monitoring the [M + Na]^+^ ion, as reported by Dall’Asta *et al*. [23].

Matrix-matched calibration curves (calibration range 100–2500 µg/kg) were used for target analyte quantification. A good linearity was obtained for all the considered mycotoxins (*r*^2^ > 0.99). For all the target compounds, limit of quantification (LOQ) and limit of detection (LOD) were lower than 30 µg/kg and 10 µg/kg, respectively.

### 3.6. Ergosterol Extraction and Analysis

Lipid fraction was extracted from wheat by stirring 1 g of milled sample in 20 mL of n-hexane for 1 h at room temperature. After that, the extract was filtered on paper filter and dried under vacuum.

Lipid fraction, containing fatty acids and sterols, were dissolved in 1 mL of hexane, added of 0.3 mL of an internal standard solution (cholestanol, 100 µg/mL) and transesterified by adding 1 mL of 5% KOH in methanol and shaking vigorously. The organic phase containing ergosterol was then purified on a silica gel cartridge eluting the fraction of interest with 3 mL of ethyl acetate, in accordance with Annaratone *et al*. [33]. The residue was dissolved in 0.5 mL of hexane, with 50 µL of BSTFA added, and reacted at 60 °C for 1 h. Analysis and quantification of ergosterol was performed by GC-MS, injecting 1 μL of the obtained solution.

GC-MS analysis was performed on a 6890N gas chromatograph coupled to a 5973N mass selective detector (Agilent technologies, Santa Clara, CA, USA), using a SLB-5MS capillary column (Supelco, Bellefonte, PA, USA). The programmed temperature gradient was as follows: 240 °C for 3 min, then increasing to 280 °C in 2 min and holding this final condition for 20 min. Injection was performed in split mode. Data acquisition mode was SIM, monitoring 460.4 and 445.4 m/z for cholestanol (internal standard) and 468.4 and 363.3 m/z for ergosterol detection.

## 4. Conclusions

The study presented herein showed for the first time the DON-to-D3G conversion ability of several durum wheat lines in comparison to Sumai-3 soft wheat under greenhouse conditions. In addition, for the first time, the D3G formation in plants already 24 h from the DON treatment and five days from the fungal infection was reported. Among the considered lines, Claudio cv. supported the highest fungal growth and the highest DON accumulation upon *F. graminearum* inoculation, showing also the lowest [D3G]/[TDON] ratio. This result was further confirmed upon DON-treatment, thus suggesting that low glycosylation ability is also related to a high susceptibility towards fungal infection and toxin accumulation, at least under greenhouse conditions. Furthermore, Kofa cv. showed a good glucosylation activity towards DON in both the assays and is a promising candidate for further studies to better define the DON detoxification activity in durum wheat.

Data here represented support thus the hypothesis reported by Lemmens *et al*. [19]: the ability to convert DON to D3G in these lines seems actually to be related to their resistance towards FHB. The role played by D3G as DON detoxification products in durum wheat was demonstrated, thus pinpointing as well the necessity for careful monitoring of this masked mycotoxin in durum wheat.
